# Changes in rhizosphere microbial communities in potted cucumber seedlings treated with syringic acid

**DOI:** 10.1371/journal.pone.0200007

**Published:** 2018-06-28

**Authors:** Zhilin Wang, Jianhui Zhang, Fengzhi Wu, Xingang Zhou

**Affiliations:** 1 Department of Horticulture, Northeast Agricultural University, Harbin, China; 2 Key Laboratory of Biology and Genetic Improvement of Horticultural Crops (Northeast Region), Ministry of Agriculture, Harbin, China; University of Calcutta, INDIA

## Abstract

Phytotoxic effects of phenolic compounds have been extensively studied, but less attention has been given to the effects of these compounds on soil microbial communities, which are crucial to the productivity of agricultural systems. Responses of cucumber rhizosphere bacterial and fungal communities to syringic acid (SA), a phenolic compound with autotoxicity to cucumber, were analyzed by high-throughput sequencing of 16S rRNA gene and internal transcribed spacer amplicons. SA at the concentration of 0.1 μmol g^-1^ soil changed rhizosphere bacterial and fungal community compositions, decreased bacterial community diversity but increased fungal community richness and diversity (P<0.05). Moreover, SA increased the relative abundances of bacterial phylum *Proteobacteria* and fungal classes *Leotiomycetes*, *Pezizomycetes*, *Tremellomycetes* and *Eurotiomycetes*, but decreased the relative abundances of bacterial phylum *Firmicutes* and fungal class *Sordariomycetes* (P<0.05). At the genus level, SA decreased the relative abundances of microbial taxa with pathogen-antagonistic and/or plant growth promoting potentials, such as *Pseudomonas* spp. (P<0.05). Real-time PCR validated that SA decreased cucumber rhizosphere *Pseudomonas* spp. abundance (P<0.05). *In vitro* study showed that SA (0.01 to 10 mM) inhibited the growth of a strain of *Pseudomonas* spp. with pathogen-antagonistic activities to cucumber pathogen *Fusarium oxysporum* f.sp. *cucumerinum* Owen (P<0.05). Overall, SA changed cucumber rhizosphere bacterial and fungal community compositions, which may exert negative effects on cucumber seedling growth through inhibiting plant-beneficial microorganisms.

## Introduction

Modern agricultural systems, which are often characterized by short-term rotation or monocropping, usually decrease the plant diversity in the field [1]. The continuous monocropping system, in which the same crop is repeatedly monocropped on the same land, is not long-term sustainable because it usually decrease crop yield and quality, a phenomenon termed as ‘soil sickness’ [1,2]. Allelopathy is the suppression of the growth of one plant by another plant through the release of toxic chemical compounds (allelochemicals) into the environment [3,4]. Autotoxicity is a type of intra-specific allelopathy, where a plant species inhibits the growth of its own kind, and is supposed to be contributed to the soil sickness associated with several crops, such as asparagus (*Asparagus officinalis* L.), cucumber (*Cucumis sativus* L.), rice (*Oryza sativa* L.), sugarcane (*Saccharum officinalis* L.) and tea (*Camellia sinensis* L.) [5–7].

As an important class of plant secondary metabolites, phenolic compounds are suggested to be involved in plant-plant interactions and plant-microorganism interactions [7–10]. Several phenolic compounds, including derivatives of benzoic and cinnamic acids, have been identified from plant root exudates and decaying residues [6,11]. Mounting evidence shows that these compounds can have phytotoxic effects at proper concentrations, such as inhibiting plant nutrient ion uptake, enzyme activities and photosynthesis and respiration [3,6].

Soil microorganisms play critical roles in terrestrial ecosystems because they have profound influences on plant health and fitness, nutrient cycling, and decomposition of organic matter [12,13]. For example, many species of *Fusarium* are phytopathogenic fungi, which can cause diseases including vascular wilts and root rots [14]; while some *Pseudomonas* spp. can inhibit several plant pathogens and promote plant growth [15]. Previous studies demonstrated that phenolic compounds can affect microbial growth, biofilm formation, virulence and expression of genes encoding secondary metabolite production [16–18]. Moreover, phenolic compounds can have selective effects on microorganisms. For example, *p*-coumaric acid promoted the growth of *F*. *oxysporum* f.sp. *cucumerinum* Owen (FOC), a soil-borne plant pathogen of cucumber [19], while ferulic acid inhibited the growths of *P*. *fluorescens* and *Glomus intraradices* [18,20]. However, information about how phenolic compounds influence the whole soil microbial communities is still limited.

Soil sickness is one of the major constraints of cucumber production in the greenhouse [21,22]. Syringic acid (SA, 4-Hydroxy-3,5-dimethoxybenzoic acid) has been identified in cucumber-cultivated soils, and had inhibitory effects on cucumber seedling growth [22,23]. In a previous study, we found that SA changed cucumber rhizosphere bacterial and fungal community structures as evaluated by PCR & Denaturing Gradient Gel Electrophoresis [23]. However, detailed changes in rhizosphere microbial compositions are still unclear. In the present, we further analyzed cucumber rhizosphere bacterial and fungal communities with high-throughput amplicon sequencing technique, which can provide a higher resolution and a better understanding of environmental microbial communities than the PCR-based fingerprinting techniques [24,25]. Cucumber seedlings were treated with SA every other day. Rhizosphere microbial communities were analyzed ten days after the treatment. Moreover, cucumber rhizosphere *Pseudomonas* spp. abundance was estimated with real-time PCR assays. The effect of SA on the growth of a strain of *Pseudomonas* spp., a bacterium antagonistic to FOC, was also evaluated *in vitro*.

## Materials and methods

### Pot experiment

Soils were collected from the upper soil layer (0–15 cm) of an open field in the experimental station of Northeast Agricultural University, Harbin, China (45°41’N, 126°37’E), which was covered with grass and undisturbed for more than 15 years. No specific permissions were required for these locations/activities and the soil sampling did not involve endangered or protected species. The soil was a Mollisol soil with sandy loam texture, contained organic matter, 3.67%; available N, 89.02 mg kg^-1^; Olsen P, 63.36 mg kg^-1^; available K, 119.15 mg kg^-1^; EC (1:2.5, w/v), 0.33 mS cm^-1^; and pH (1:2.5, w/v), 7.78.

Cucumber seedlings (cv. Jinlv 3) with two cotyledons were planted into pots (10 cm diameter, 10 cm height) contained 150 g of soil. No fertilizer was used during the experiment. There was one cucumber seedling per pot. Cucumber seedlings were maintained in a greenhouse (32°C day/22°C night, relative humidity of 60–80%, 16 h light/8 h dark).

Since phenolic acids could be rapidly metabolized by soil microorganisms after entering the soil [26], SA was applied into the soil periodically as suggested by Blum *et al*. [27]. At the one-leaf stage, cucumber seedlings were treated with SA at the concentration of 0.1 μmol g^-1^ soil every two days for five times. Seedlings treated with distilled water were used as the control. The solution pH was adjusted to 7.0 with 0.1 M sodium hydroxide solution because the soil pH is an important factor that regulates soil microbial communities [28]. Each treatment had five pots and was done in triplicate. Soil water content was adjusted every two days with distilled water to maintain the soil moisture at about 60% of its water holding capacity.

### Soil sampling and DNA extraction

Ten days after the first treatment of SA, cucumber rhizosphere soils were collected from five seedlings in each replicate and were mixed to make a composite sample as described before [23]. There were three composite samples for each treatment and six composite samples in total.

Total soil DNA was extracted from 0.25 g soil with the PowerSoil DNA Isolation Kit (MO BIO Laboratories, Carlsbad, USA) according to the manufacturer’s instructions. Each composite soil sample was extracted in triplicate and the extracted DNA solutions were pooled. There were three composite DNA samples for each treatment.

### Illumina Miseq sequencing and data processing

Cucumber rhizosphere bacterial and fungal community compositions were analyzed with high-throughput amplicon sequencing. The V3-V4 regions of the bacterial 16S rRNA gene and the ITS1 regions of the fungal rRNA gene were amplified with primer sets of F338/R806 and ITS1F/ITS2, respectively, as described before [29–31]. Both the forward and reverse primers also had a 6-bp barcode unique to each sample. Each DNA sample was independently amplified in triplicate, and the products of the triplicate PCR reactions were pooled and purified using the Agarose Gel DNA purification kit (TaKaRa, China). Then, purified amplicons were quantified by a TBS-380 micro fluorometer with Picogreen reagent (Invitrogen, USA), and mixed accordingly to achieve the equal concentration in the final mixture. The mixture was then paired-end sequenced (2×300) on an Illumina Miseq platform at Majorbio Bio-Pharm Technology Co., Ltd., Shanghai, China.

Raw sequence reads were de-multiplexed, quality-filtered, and processed using FLASH as described before [31,32]. Chimeric sequences were identified and removed using USEARCH 6.1 in QIIME [33]. Sequences were binned to Operational taxonomic units (OTUs) at 97% sequence similarity with USEARCH using an agglomerative clustering algorithm [34]. Then, a representative sequence of each OTU was taxonomically classified through BLAST against the SILVA [35] (bacteria) and Unite [36] (fungi) databases. The data set was deposited in the NCBI-Sequence Read Archive with the submission Accession Number SRP115338.

### Real-time PCR analysis

Cucumber rhizosphere *Pseudomonas* spp. abundance was estimated with SYBR Green real-time PCR assays with primers of PsF/PsR [37] on an IQ5 real-time PCR system (Bio-Rad Lab, USA). The PCR protocol was: 95°C for 5 min; followed by 30 cycles of 95°C for 30 s, 65°C for 30 s, 72°C for 90 s; and a final extension at 72°C for 10 min. Standard curves were made with a 10-fold dilution series (10^2^−10^8^) of plasmids containing 16S rRNA genes from soil samples. Sterile water was used as a negative control to replace the DNA template. All amplifications were done in triplicate. The specificity of the products was confirmed by melting curve analysis and agarose gel electrophoresis. The threshold cycle (*Ct*) values obtained for each sample were compared with the standard curve to determine the initial copy number of the target gene.

### *In vitro* experiment

Effects of SA on the growth of *Pseudomonas* spp. was studied *in vitro* with a microtiter plate-based assay method [38]. A strain of *Pseudomonas* spp. with antagonistic activity to FOC, *Pseudomonas* ZJH, was isolated and identified from greenhouse soils cultivated with tomato. *Pseudomonas* ZJH was grown aerobically in Luria-Bertani (LB) broth at 37°C with shaking at 120 rpm overnight (in the log phase of growth). The culture was diluted to a final concentration of 1×10^6^ cfu/ml with fresh LB medium supplemented with 0, 0.01, 0.1, 0.5, 1.0 and 10 mM of SA in 100 ml flasks. Then, the bacterial suspension was transferred into 96-well polystyrene microtiter plates with each well containing 200 μl of bacterial suspension. Microtiter plates were incubated at 37°C with shaking at 120 rpm and the optical density at 600 nm (OD_600_) in the wells was measured using a microtitre plate reader (Epoch, Biotek, USA) at 16 h. There were three microtiter wells for each treatments and the experiment was done in triplicate.

### Statistical analysis

To avoid potential bias caused by sequencing depth, a random subsampling effort of 21,334 16S rRNA gene and 30,394 ITS gene sequences per sample was performed. The defined OTUs were used to calculate taxon accumulation curves with the ‘vegan’ package in ‘R’ (Version 3.3.1). For the alpha diversity analysis, Chao, ACE, Shannon index and inverse Simpson index were calculated using QIIME [33]. For the beta diversity analysis, weighted UniFrac distances and Bray-Curtis distances were calculated using QIIME [33] and ‘vegan’ package in ‘R’ (Version 3.3.1), respectively. Principal coordinates analysis was conducted to visualize the community similarity with the ‘vegan’ package in ‘R’ (Version 3.3.1). The shared and unique OTUs among treatments were counted, and their distributions were shown in a Venn diagram with the ‘VennDiagram’ package in ‘R’ (Version 3.3.1).

Data were analyzed by analysis of variance (ANOVA) in ‘R’ (Version 3.3.1). For data of alpha diversity indices and relative abundances of microbial taxa from Illumina Miseq sequencing analysis, and *Pseudomonas* spp. abundance from real-time PCR analysis, mean comparison between treatments was performed based on Welch’s *t* test at the 0.05 probability level. For data of the growth of *Pseudomonas* spp. from the *in vitro* experiment, mean comparison between treatments was performed based on the Tukey’s honestly significant difference (HSD) test at the 0.05 probability level.

## Results

### Sequence summary

After filtering reads by basal quality control and removing singleton OTUs, Illumina Miseq sequencing generated 24,696 quality bacterial sequences and 38,658 quality fungal sequences per sample on average. The average read lengths were 396 bp and 261 bp for the 16S rRNA genes and ITS regions, respectively. After clustering at the 97% sequence similarity, 1947 and 339 OTUs were identified for bacterial and fungal communities, respectively.

The Good’s coverage of each soil sample, which reflects the captured diversity, was higher than 98% for bacterial community and higher than 99% for fungal community (data not shown). Rarefaction curves of OTUs at the 97% sequence similarity of all samples tended to approach the saturation plateau (S1 Fig), which also indicated that the sequencing depth was adequate.

### Alpha and beta diversities of bacterial and fungal communities

**Fig 1:**
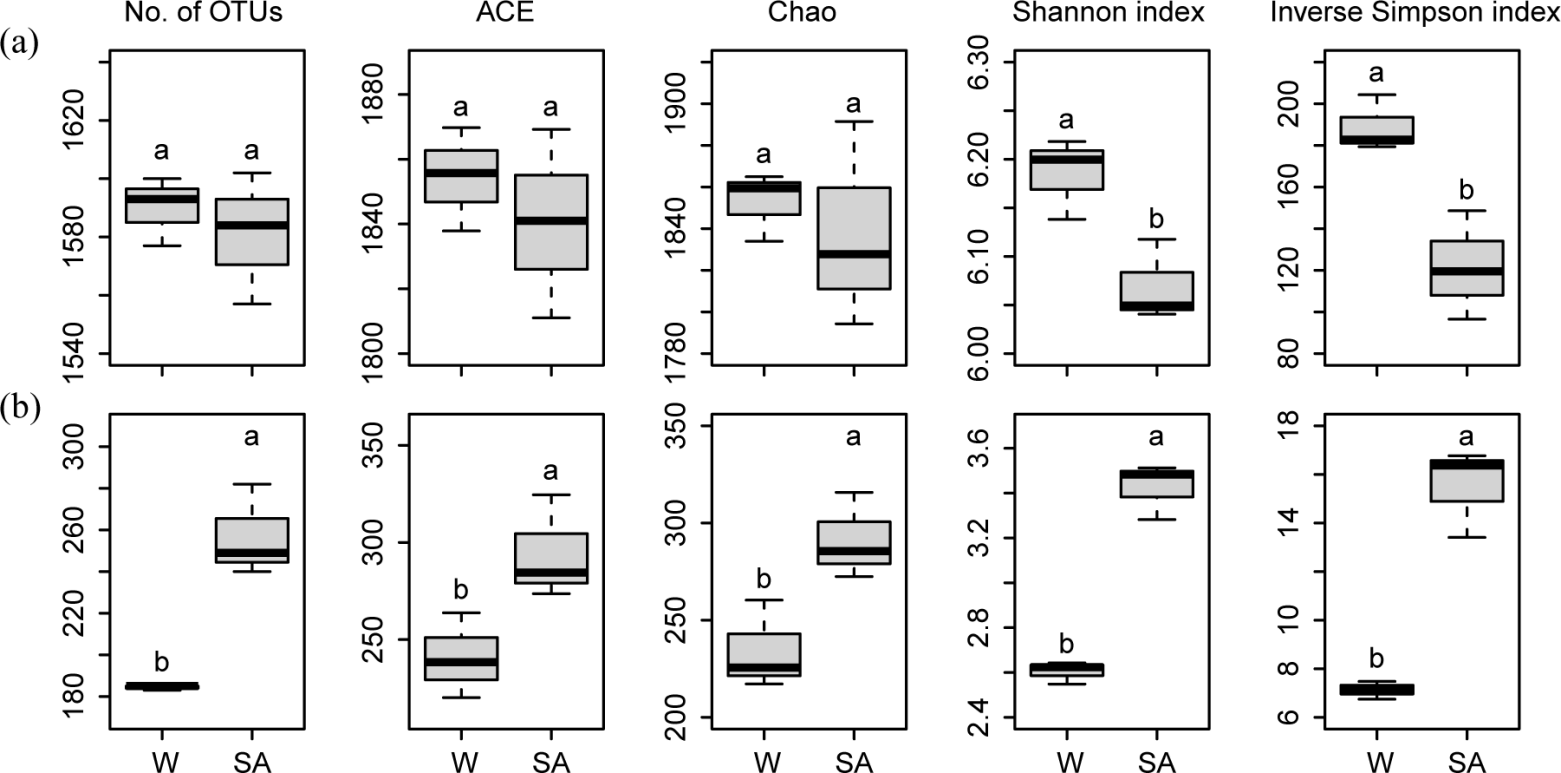
**Diversity and richness indices of soil bacterial (a) and fungal (b) communities.** OTUs were delineated at the 97% sequence similarity. These indices were calculated using random subsamples of 21,334 16S rRNA gene and 30,394 ITS gene sequences per sample. Different letters indicate significant difference based on Welch’s *t* test (P<0.05). SA and W represent syringic acid- and water-treated soil samples, respectively.

For bacterial communities, the number of OTUs, ACE and Chao indices did not significantly differ between the SA- and water-treated soil samples (Fig 1A). However, the Shannon index and inverse Simpson index were significantly lower in the SA-treated soil sample than in the water-treated soil sample (P<0.05).

For fungal communities, the number of OTUs, ACE, Chao, Shannon, and inverse Simpson indices were significantly higher in the SA-treated soil sample than in the water-treated soil sample (P<0.05) (Fig 1B).

**Fig 2:**
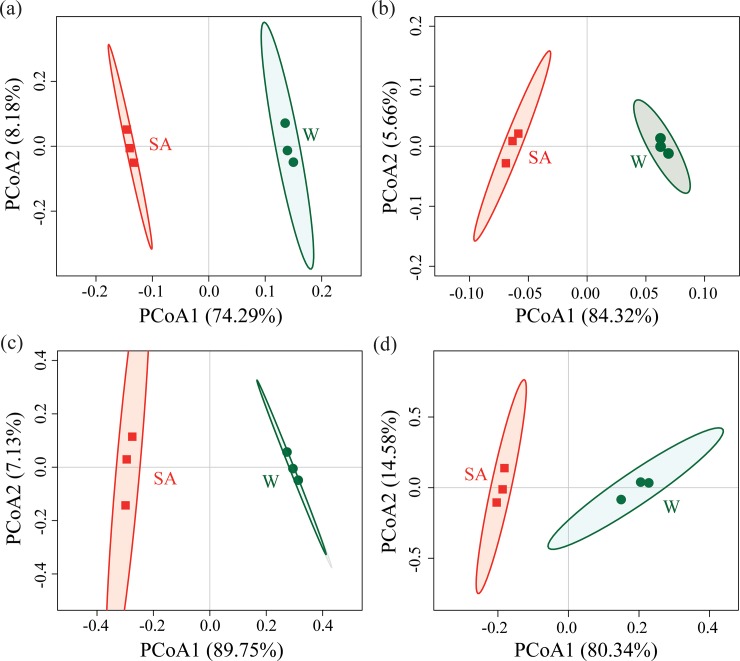
Beta diversities of bacterial and fungal communities. Differences in Bray-Curtis (a) and UniFrac distances (b) of bacterial communities, Bray-Curtis (c) and UniFrac distances (d) of fungal communities were visualized by principal component analyses. SA and W represent syringic acid- and water-treated soil samples, respectively. Ellipses indicate 95% confidence interval for replicates.

For both bacterial and fungal communities, principal coordinates analysis based on Bray-Curtis and UniFrac distances resulted in a clear separation of SA- and water-treated soil samples along the first axis (Fig 2).

### Bacterial community composition

**Fig 3:**
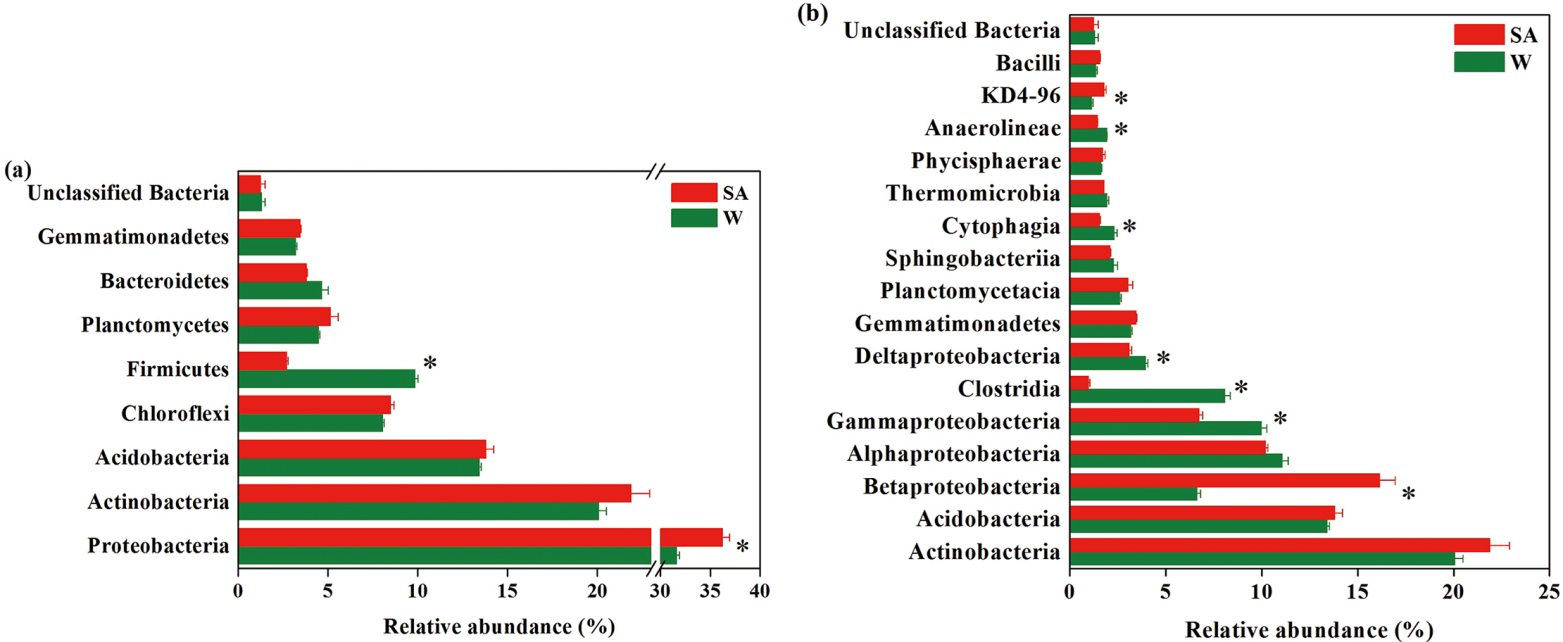
**Relative abundances of main bacterial phyla (a) and classes (b) in the syringic acid (SA)- and water (W)-treated soil samples.** Bacterial phyla and classes with average relative abundances >1% in at least one treatment were shown. Values are expressed as mean±standard error. Asterisks indicate significant difference between treatments based on Welch’s *t* test (P<0.05).

Across all samples analyzed, a total of 27 bacterial phyla were detected, and 1.28% bacterial sequences were unclassified at the phylum level. The top three bacterial phyla were *Proteobacteria*, *Actinobacteria* and *Acidobacteria*, which had relative abundances ranging from 31.06% to 37.32%, from 19.28% to 23.27%, and from 12.96% to 14.37%, respectively (Fig 3A). These three bacterial phyla accounted for more than 68% of the bacterial sequences. *Chloroflexi*, *Firmicutes*, *Planctomycetes*, *Bacteroidetes* and *Gemmatimonadetes* were also detected at relatively high abundances (average relative abundance>1%). Compared with the water-treated soil sample, the SA-treated soil sample had significantly higher relative abundance of *Proteobacteria* and lower relative abundance of *Firmicutes* (P<0.05).

At the class level, all samples were dominated by *Actinobacteria*, *Acidobacteria*, *Betaproteobacteria*, *Alphaproteobacteria* and *Gammaproteobacteria* (average relative abundance>5%) (Fig 3B). These five bacterial classes accounted for more than 64% of the bacterial sequences. Compared with the water-treated soil sample, the SA-treated soil sample had significantly higher relative abundances of *Betaproteobacteria* and *KD4-96*, but had lower relative abundances of *Gammaproteobacteria*, *Clostridia*, *Deltaproteobacteria*, *Cytophagia* and *Anaerolineae* (P<0.05).

**Fig 4:**
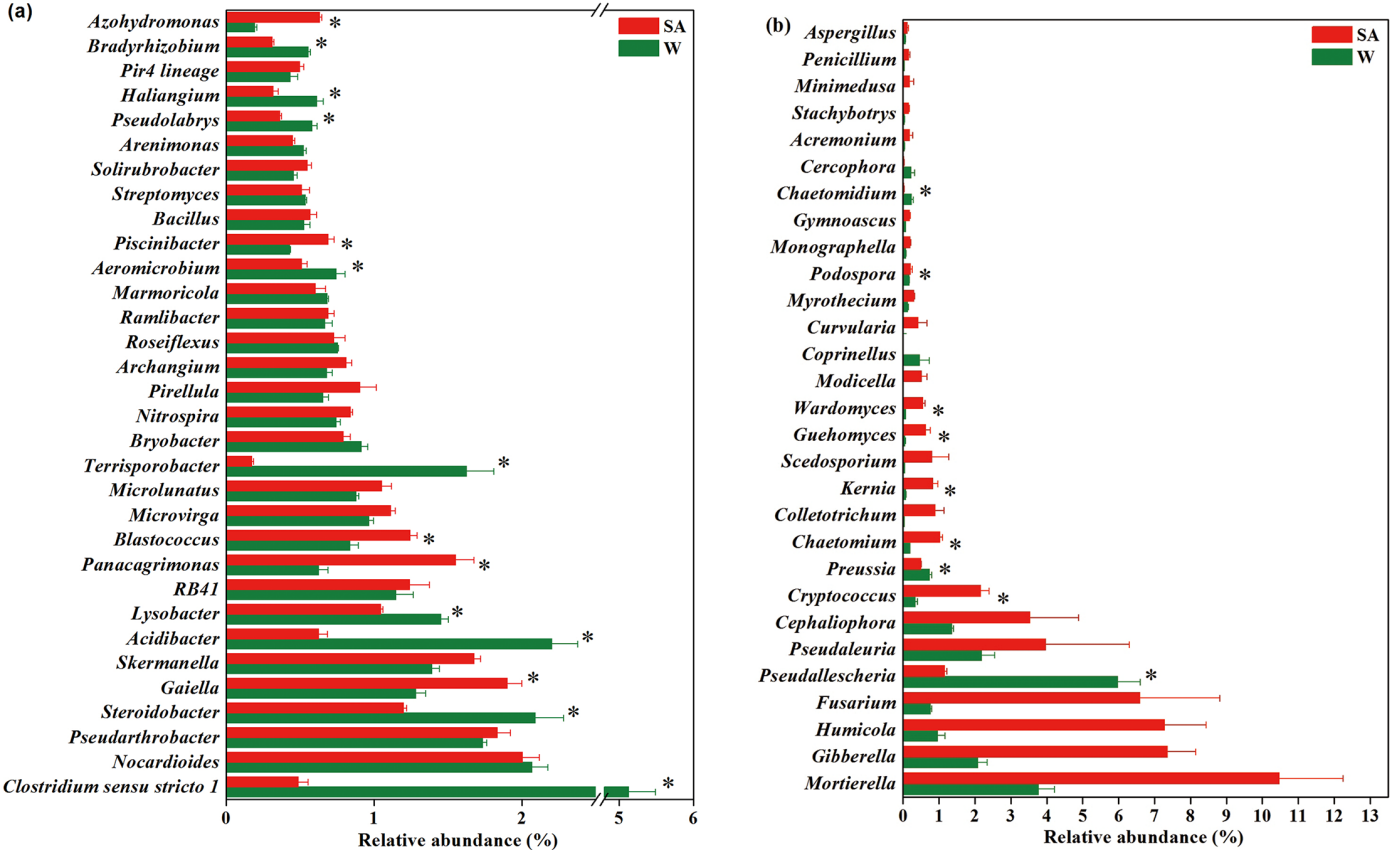
**Relative abundances of main classified bacterial (a) and fungal genera (b) in the syringic acid (SA)- and water (W)-treated soil samples.** Classified bacterial and fungal genera with average relative abundances >0.5% and 0.1%, respectively, in at least one treatment were shown. Values are expressed as mean±standard error. Asterisks indicate significant difference between treatments based on Welch’s *t* test (P<0.05).

**Fig 5:**
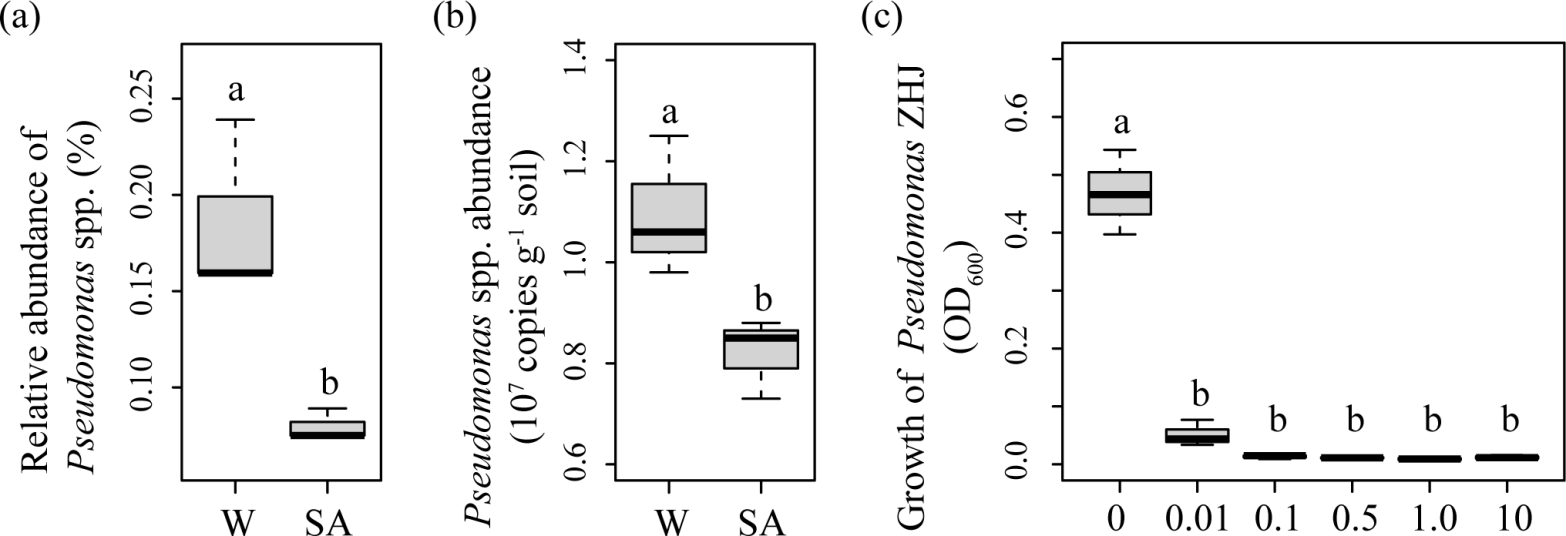
**Effects of SA on the relative abundance of *Pseudomonas* spp. as estimated by Illumina Miseq sequencing (a), the abundance of *Pseudomonas* spp. as estimated by real-time PCR (b) and the growth of *Pseudomonas* ZJH *in vitro* (c).** For (a) and (b), SA and W represent syringic acid- and water-treated soil samples, respectively. Different letters indicate significant difference based on Welch’s *t* test (P<0.05). For (c), 0, 0.01, 0.1, 0.5, 1.0 and 10 represent the treatments of 0, 0.01, 0.1, 0.5 1.0 and 10 mM of syringic acid, respectively. Different letters indicate significant difference based on Tukey’s HSD test (P<0.05).

At the genus level, compared with the water-treated soil sample, the SA-treated soil sample had significantly higher relative abundances of *Gaiella*, *Panacagrimonas*, *Blastococcus*, *Piscinibacter* and *Azohydromonas* spp., but had lower relative abundances of *Clostridium sensu stricto 1*, *Steroidobacter*, *Acidibacter*, *Lysobacter*, *Terrisporobacter*, *Aeromicrobium*, *Pseudolabrys*, *Haliangium*, *Pseudomonas* and *Bradyrhizobium* spp. (P<0.05) (Figs 4A and 5A).

At the OTU level, the relative abundances of 12 dominant OTUs (average relative abundance>0.5%) were higher while 11 dominant OTUs were lower in SA-treated soil sample than in the water-treated soil sample (P<0.05) (S1 Table). These changed dominant OTUs mainly belonged to bacterial classes *Actinobacteria*, *Clostridia*, *Alphaproteobacteria*, *Betaproteobacteria*, *Gammaproteobacteria* and *Acidobacteria*.

### Fungal community composition

**Fig 6:**
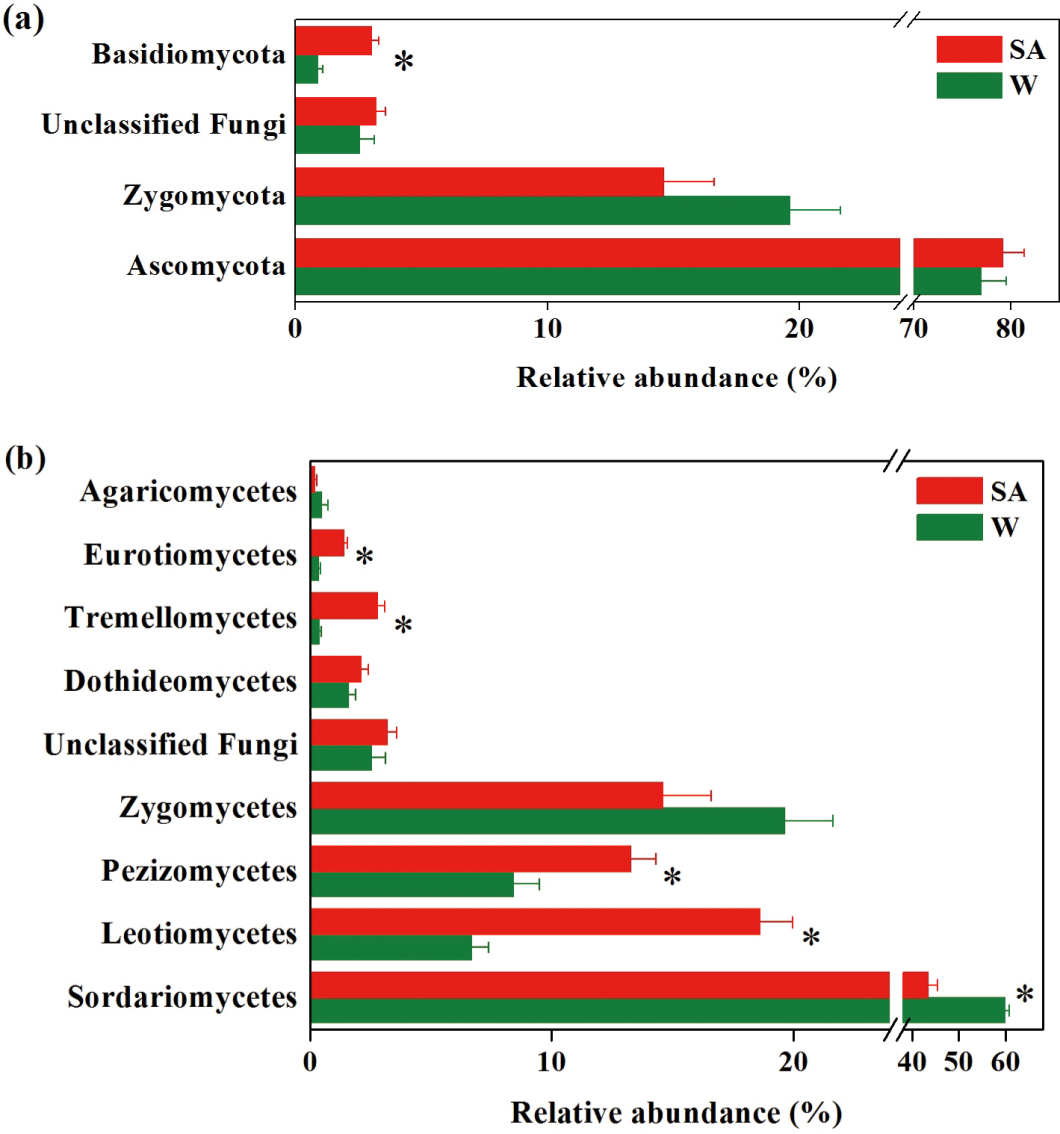
**Relative abundances of main fungal phyla (a) and classes (b) in the syringic acid (SA)- and water (W)-treated soil samples.** Fungal phyla and classes with average relative abundances >0.5% in at least one treatment were shown. Values are expressed as mean±standard error. Asterisks indicate significant difference between treatments based on Welch’s *t* test (P<0.05).

Fungal community was almost entirely composed of *Ascomycota* and *Zygomycota*, accounting for more than 95% of the fungal sequences (Fig 6A). Less abundant fungal phyla detected were *Basidiomycota* and *Chytridiomycota*. About 2.87% fungal sequences were not assigned to any known phylum. *Sordariomycetes*, *Leotiomycetes*, *Pezizomycetes* and *Zygomycetes* were the top four fungal classes, and they accounted for more than 92% of the fungal sequences (Fig 6B). Compared with the water-treated soil sample, the SA-treated soil sample had significantly higher relative abundances of phylum *Basidiomycota*, and classes *Leotiomycetes*, *Pezizomycetes*, *Tremellomycetes* and *Eurotiomycetes*, but had lower relative abundance of class *Sordariomycetes* (P<0.05).

The dominant families (average relative abundance>10%) in the SA-treated soil sample were *Chaetomiaceae* (18.91%), *Thelebolaceae* (18.55%), *Nectriaceae* (14.29%) and *Mortierellaceae* (14.08%), while these in the water-treated soil sample were *Chaetomiaceae* (31.32%), *Mortierellaceae* (19.62%) and *Lasiosphaeriaceae* (18.38%) (S2 Fig). More sequences were unclassified at the genus level in the water-treated soil sample than in the SA-treated soil sample for families *Chaetomiaceae* (29.93% vs. 10.55%), *Lasiosphaeriaceae* (17.97% vs. 3.25%) and *Mortierellaceae* (15.85% vs. 3.61%). Compared with the water-treated soil sample, the SA-treated soil sample had significantly higher relative abundances of families *Thelebolaceae*, *Tremellales incertae sedis*, *Gymnoascaceae*, and genera *Cryptococcus*, *Chaetomium*, *Kernia*, *Guehomyces* and *Wardomyces* spp., but had lower relative abundances of families *Chaetomiaceae* and *Lasiosphaeriaceae*, and genera *Pseudallescheria*, *Preussia* and *Chaetomidium* spp. (P<0.05) (Fig 4B and S2 Fig).

At the OTU level, the relative abundances of seven dominant OTUs (average relative abundance>0.5%), mainly composed of *Sordariomycetes* and *Zygomycetes*, were lower in SA-treated soil sample than in the water-treated soil sample (P<0.05) (S2 Table). The relative abundances of 27 dominant OTUs, mainly belonging to *Dothideomycetes*, *Eurotiomycetes*, *Leotiomycetes*, *Sordariomycetes*, *Tremellomycetes* and *Zygomycetes*, were higher in SA-treated soil sample than in the water-treated soil sample (P<0.05).

### Shared and unique OTUs

**Fig 7:**
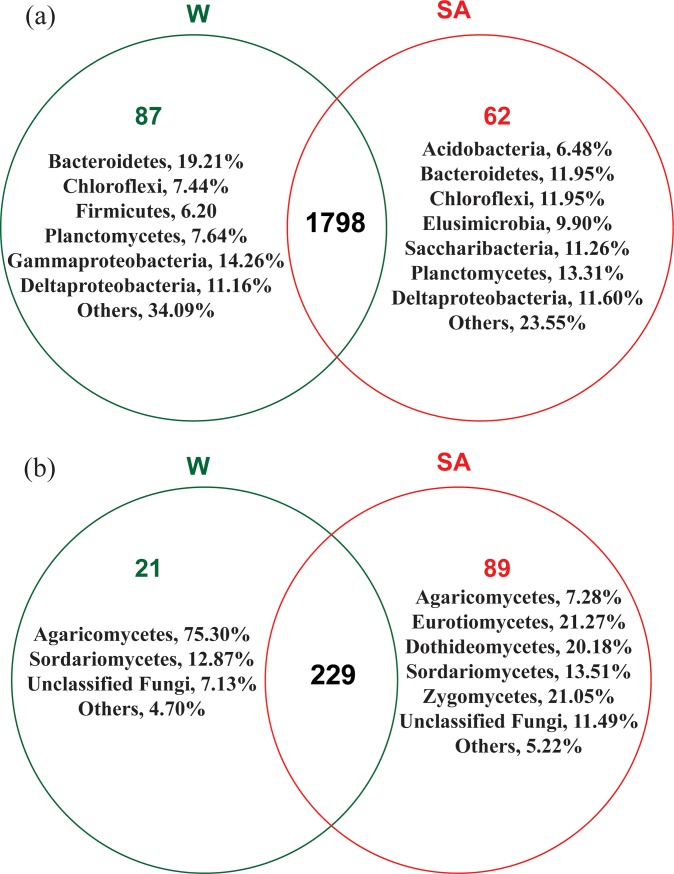
**Venn diagrams demonstrating the numbers of shared and unique observed bacterial (a) and fungal (b) OTUs at 97% similarity between syringic acid (SA)- and water (W)-treated soil samples.** Frequencies of OTUs unique to each treatment at the bacterial phylum/proteobacterial class and fungal class level were shown.

For bacterial communities, SA- and water-treated soil samples shared 1798 OTUs, which accounted for 92.35% of the total bacterial OTUs observed (Fig 7A). Only a small proportion of OTUs were found to be unique to SA- or water-treated soil samples. OTUs unique to water-treated soil sample were dominated by sequences belonging to classes *Bacteroidetes*, *Gammaproteobacteria* and *Deltaproteobacteria*. OTUs unique to SA-treated soil sample were dominated by sequences belonging to classes *Planctomycetes*, *Bacteroidetes*, *Chloroflexi*, *Deltaproteobacteria* and *Saccharibacteria*.

For fungal communities, about 67.55% of total fungal OTUs were shared by all treatments. OTUs unique to water-treated soil sample were mainly composed of sequences belonging to *Agaricomycetes* at the class level and *Coprinellus* spp. at the genus level (72.70%); while these unique to SA-treated soil sample were dominated by sequences belonging to *Eurotiomycetes*, *Dothideomycetes* and *Zygomycetes* at the class level and *Modicella* (20.48%), Unclassified *Onygenaceae* (17.63%) and *Curvularia* spp. (15.61%) at the genus level (Fig 7B).

### Cucumber rhizosphere *Pseudomonas* spp. abundance

Real-time PCR analysis showed that SA significantly decreased cucumber rhizosphere *Pseudomonas* spp. abundance (Fig 5B). The abundance of *Pseudomonas* spp. in the water-treated soil sample was about 1.34 times of that in the SA-treated soil sample.

### Effects of SA on *Pseudomonas* ZJH *in vitro*

*In vitro* experiment showed that SA at the concentrations of 0.01 to 10 mM significantly inhibited the growth of *Pseudomonas* ZJH, which was antagonistic to the soil pathogen of cucumber FOC (Fig 5C). However, the effects of SA on the growth of *Pseudomonas* ZJH did not differ among different concentrations of SA.

## Discussion

Autotoxicity is commonly acknowledged as one of the contributing factors to soil sickness, which severely threats sustainable agricultural production [6,22]. Soil microbial communities have profound effects on the growth, nutrition and health of plants in agricultural ecosystems [13]. In this study, we focused on the responses of rhizosphere microbial communities to SA, a phenolic compound with autotoxicity from cucumber. In natural ecosystems, soil SA concentration was shown to range from 0.05 to 0.1 μmol g^-1^ soil [39]. Previously, we also found that soil SA concentration in cucumber monocropping system ranged from 0.1 to 0.15 μmol g^-1^ soil [22] and exogenous SA at the concentrations of 0.05 to 0.2 μmol g^-1^ soil have detrimental effects on cucumber seedling growth [23]. Therefore, SA at 0.1 μmol g^-1^ soil was used in this study to simulate its effects on cucumber rhizosphere microbial communities in the monocropping system.

In the present study, the diversity indices of the bacterial communities (Shannon and inverse Simpson indices) were lower while these of the fungal communities were higher in the SA-treated soil sample than in the water-treated soil sample (P<0.05). Previously, our real-time PCR analysis showed that exogenous SA stimulated both bacterial and fungal community abundances with fungal community dad a larger increase [23]. These results suggested that bacterial and fungal communities responded differently to exogenous SA. This is not surprising considering that soil bacteria and fungi play different roles in degrading organic compounds. For example, fungi are generally regarded as main lignocellulose decomposers while bacteria prefer easily available carbon sources [40]. It has been observed that different microbial species have different abilities to degrade phenolic acids and also differ in their tolerances to the toxicity of phenolic acids [17,41,42]. Antagonistic interactions, through competition for substrate and production of antibiotics, occur between soil microorganisms [43]. The increased fungal community diversity may be due to its less antagonism with bacterial community.

Principal coordinates analysis of the high-throughput amplicon sequencing data revealed that SA changed cucumber rhizosphere bacterial and fungal community structure, which confirmed our previous results of DNA fingerprinting analysis [23]. Our results also showed that the relative abundances of some taxa were higher while others were lower in SA-treated soil sample than in the water-treated soil sample, which was consistent with previous observations showing that plant root-released compounds had selective effects on soil microorganisms by promoting certain microorganisms and inhibiting others [44–46]. For example, phenolic compounds have been shown to promote the growth of *Fusarium* spp. [19,47] but inhibit the growth of *Azohydromonas* and *Pseudomonas* spp. at proper concentrations *in vitro* [48].

Microorganisms in soils are usually limited by the availability of carbon resources [12,44,49,50]. After entering soil, plant root released compounds can be assimilated by soil microorganisms [49]. In accordance with this notion, our SA-treated soil sample were enriched with several microbial taxa that were reported to be able to degrade simple phenolic compounds or other organic compounds containing aromatic rings, such as *Azohydromonas* [51], *Blastococcus* [52], *Chaetomium* [53], *Cryptococcus* [54] and *Guehomyces* [55] spp. Further studies should focus on validating the role of these microorganisms in degrading SA with techniques such as stable isotope probing.

Soil microorganisms can affect plant growth directly through forming mutualistic and pathogenic interactions, and indirectly through enhancing nutrient cycling as free-living ones [12,13]. Our Illumina Miseq sequencing results showed that SA-treated soil sample had significantly lower relative abundances of microbial taxa with plant-growth promoting potentials than in the water-treated soil sample. For example, species of *Pseudomonas* are known for their abilities to promote plant growth through phosphate solubilization and the production of phytohormones and volatile growth stimulants [56]. *Aeromicrobium* spp. can produce indole-3-acetic acid (IAA) and solubilize phosphate [57]. *Bradyrhizobium* spp. contains strains with high phosphate solubilizing, IAA and siderophore producing activities, and can promote the growth of radish (*Raphanus sativus* L.) [58]. Some *Preussia* spp. have plant growth-promoting activities through releasing IAA [59,60].

Microbial taxa that had plant pathogen-inhibiting potentials or may be related to soil suppressiveness to soil-borne pathogens were inhibited by SA. For example, *Haliangium*, *Lysobacter*, *Pseudomonas* and *Preussia* spp. can produce secondary metabolites to inhibit plant fungal pathogens [61,62]. Species of *Chaetomidium* spp. inhibited the growth of *Gaeumannomyces graminis* var. *tritici*, a root pathogen of barley [63]. *Aeromicrobium* spp. was higher in potato common scab-suppressive soil [64]. *Steroidobacter* spp. was higher in the rhizosphere of healthy *Lilium davidii* var. *unicolor* than in the *Fusarium*-wilted ones [65]. Soil that was suppressive to Panama disease of banana had higher *Pseudolabrys* spp. [66]. Both Illumina Miseq sequencing and real-time PCR showed that SA had inhibitory effect on *Pseudomonas* spp. in cucumber rhizosphere. *In vitro* experiment also confirmed that SA inhibited the growth of a strain of *Pseudomonas* spp. with antagonistic activity to FOC. Therefore, the inhibition of plant beneficial microbes by phenolic compounds may be linked to the retarded plant growth and accumulation of soil-borne plant pathogens that observed in long-term monocropping systems [1,2,31].

## Conclusions

In summary, we showed that SA changed cucumber rhizosphere bacterial and fungal community compositions, decreased the bacterial community diversity but increased fungal community richness and diversity. Moreover, SA have selective effects on soil microorganisms by promoting certain microorganisms and inhibiting others. Importantly, SA decreased the relative abundances of several microbial taxa with plant-growth promoting or plant pathogen-inhibiting potentials. Real-time PCR analysis and *in vitro* experiment validated that SA had inhibitory effects on *Pseudomonas* spp. Plant-released compounds play an important role in the complex interactions that occur between plants and soil microorganisms, and among soil microorganisms [46,49,50]. Further investigations are required to elucidate the interactions among cucumber, *Pseudomonas* spp. and soil-borne pathogens (e.g., FOC) mediated by SA.

## Supporting information

S1 Fig **Rarefaction curves of the number of operational taxonomic units (OTUs) for bacterial (a) and fungal communities (b) in each sample.** Random subsamples of 21,334 16S rRNA gene and 30,394 ITS gene sequences per sample were used to generate the rarefaction curves. OTUs were delineated at the 97% sequence similarity.
(TIF)

S2 Fig Relative abundances of fungal families in the syringic acid (SA)- and water (W)-treated soil samples. Fungal families with average relative abundances >1% (a) and >0.1% (b) in at least one treatment were shown. Values are expressed as mean±standard error. Asterisks indicate significant difference between treatments based on Welch’s *t* test (P<0.05).
(TIF)

S1 Table The most abundant bacterial OTUs in the syringic acid (SA)- and water (W)-treated soil samples. OTUs were delineated at the 97% sequence similarity. Only OTUs with average relative abundances >0.5% in at least one treatment were presented. Values were expressed as mean±standard error. OTU ID in bold indicates its relative abundance was significant different between treatments according to Welch’s *t* test (P<0.05).
(DOC)

S2 Table The most abundant fungal OTUs in the syringic acid (SA)- and water (W)-treated soil samples. OTUs were delineated at the 97% sequence similarity. Only OTUs with average relative abundances >0.1% in at least one treatment were presented. Values were expressed as mean±standard error. OTU ID in bold indicates its relative abundance was significant different between treatments according to Welch’s *t* test (P<0.05).
(DOC)
