# Supplementary figures and images for: Changes in rhizosphere microbial communities in potted cucumber seedlings treated with syringic acid
Source: PLoS One. 2018 Jun 28;13(6):e0200007. doi: 10.1371/journal.pone.0200007 (PMC6023137; doi:10.1371/journal.pone.0200007)

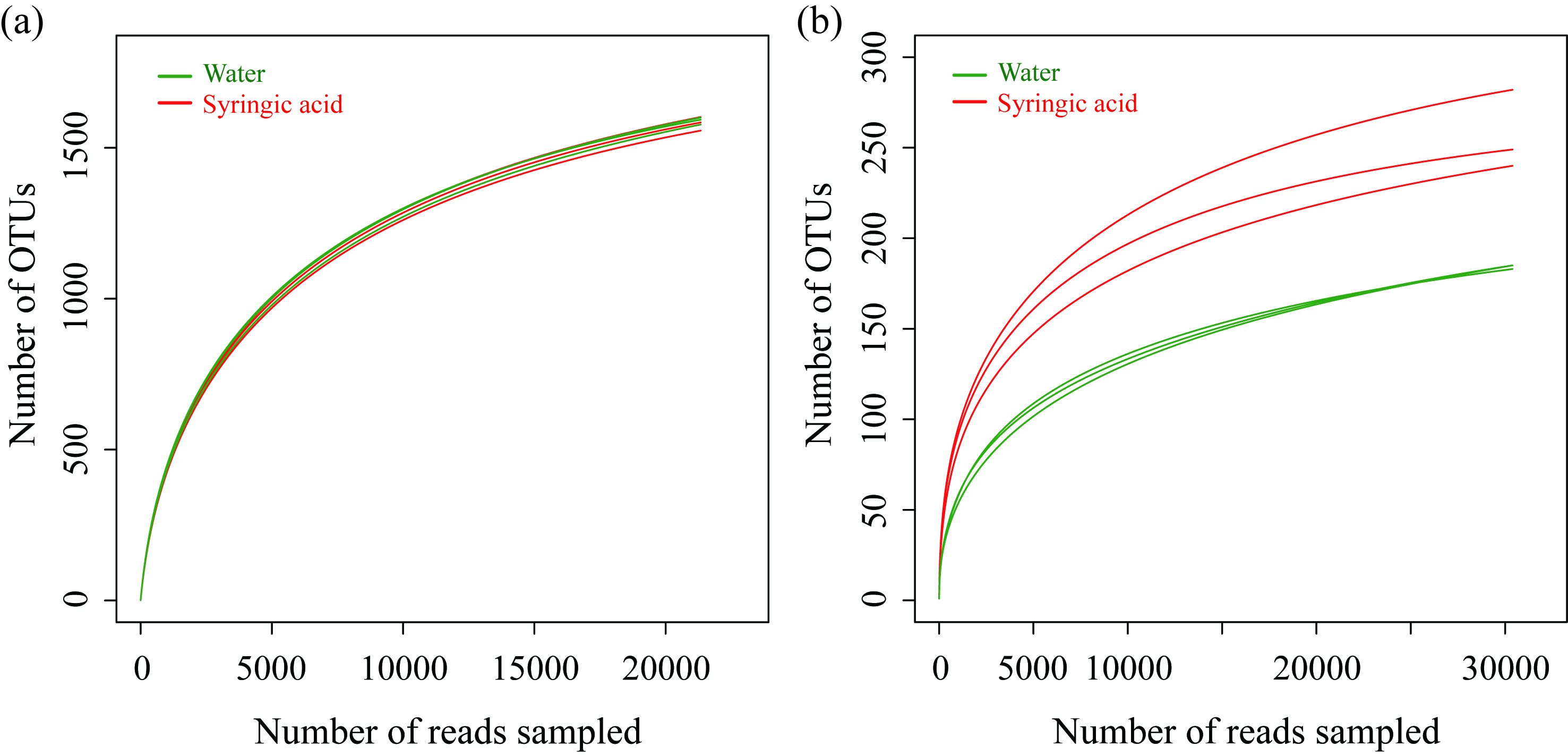

Supplement: S1 Fig — Rarefaction curves of the number of operational taxonomic units (OTUs) for bacterial (a) and fungal communities (b) in each sample. Random subsamples of 21,334 16S rRNA gene and 30,394 ITS gene sequences per sample were used to generate the rarefaction curves. OTUs were delineated at the 97% sequence similarity. (TIF) [file pone.0200007.s001.tif]

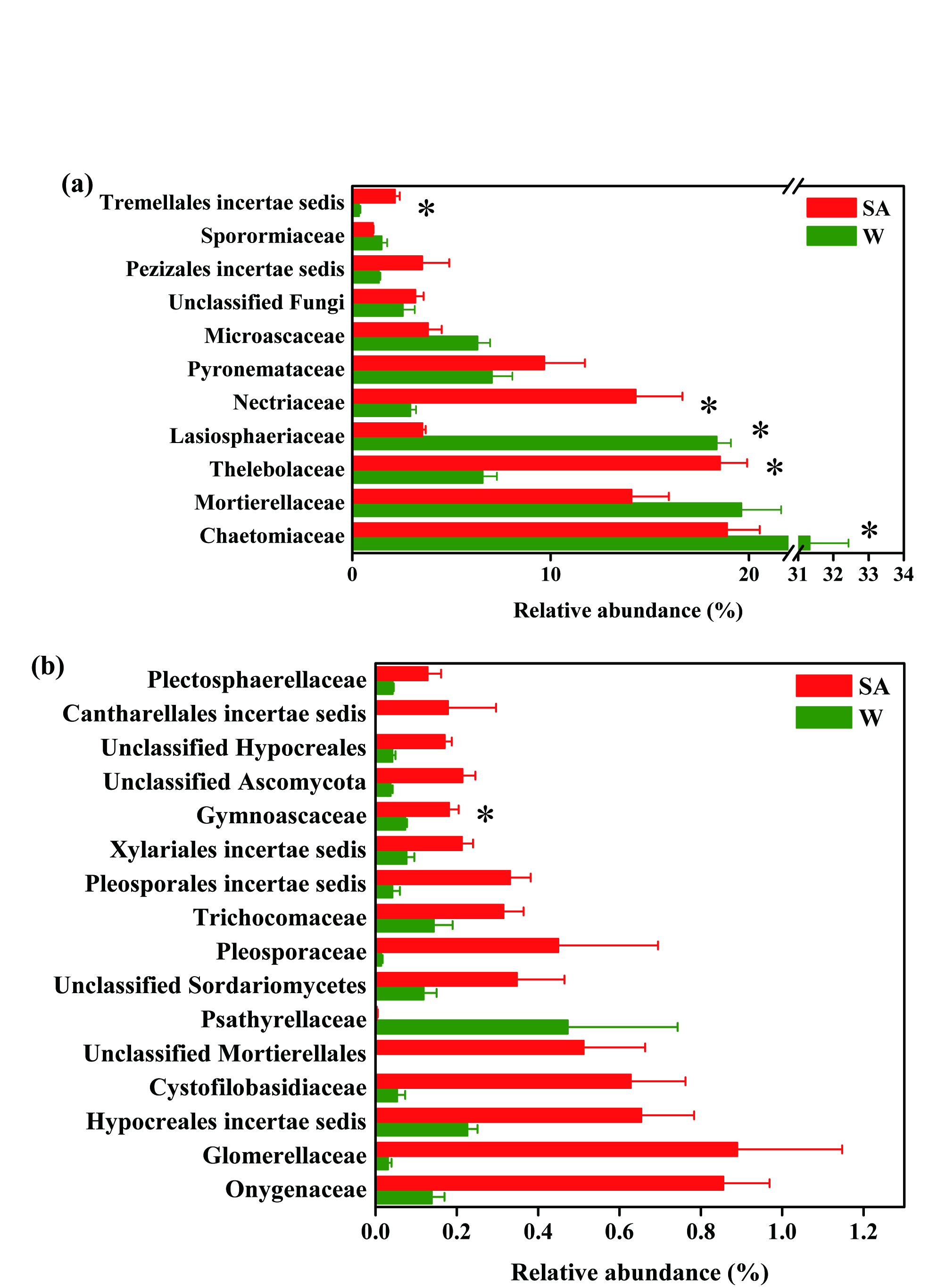

Supplement: S2 Fig — Fungal families with average relative abundances >1% (a) and >0.1% (b) in at least one treatment were shown. Values are expressed as mean±standard error. Asterisks indicate significant difference between treatments based on Welch’s t test (P<0.05). (TIF) [file pone.0200007.s002.tif]
