# Supplementary material for: Changes in rhizosphere microbial communities in potted cucumber seedlings treated with syringic acid
Source: PLoS One. 2018 Jun 28;13(6):e0200007. doi: 10.1371/journal.pone.0200007 (PMC6023137; doi:10.1371/journal.pone.0200007)
Supplement: S1 Table — OTUs were delineated at the 97% sequence similarity. Only OTUs with average relative abundances >0.5% in at least one treatment were presented. Values were expressed as mean±standard error. OTU ID in bold indicates its relative abundance was significant different between treatments according to Welch’s t test (P<0.05). (DOC) [file pone.0200007.s003.doc]

**Table S1.** **The most abundant bacterial OTUs in the syringic acid (SA)- and water (W)-treated soil samples.** OTUs were delineated at the 97% sequence similarity. Only OTUs with average relative abundances >0.5% in at least one treatment were presented. Values were expressed as mean±standard error. OTU ID in bold indicates its relative abundance was significant different between treatments according to Welch’s *t* test (P<0.05).

| OTU ID | Phylum | Class | Genus/Species | Relative abundances (%) | |
| --- | --- | --- | --- | --- | --- |
|  |  |  |  | W | SA |
| **OTU1769** | Firmicutes | Clostridia | *Clostridium sensu stricto 1* | 3.90±0.27 | 0.37±0.04 |
| OTU1064 | Actinobacteria | Actinobacteria | *Pseudarthrobacter* | 1.73±0.03 | 1.83±0.09 |
| OTU1407 | Acidobacteria | Acidobacteria | Acidobacteria norank | 1.35±0.04 | 1.48±0.05 |
| OTU1767 | Proteobacteria | Gammaproteobacteria | *Steroidobacter* | 1.64±0.17 | 1.05±0.03 |
| **OTU1308** | Proteobacteria | Gammaproteobacteria | *Acidibacter* | 1.88±0.18 | 0.39±0.04 |
| **OTU1191** | Proteobacteria | Gammaproteobacteria | *Panacagrimonas* | 0.62±0.07 | 1.55±0.13 |
| **OTU1737** | Actinobacteria | Actinobacteria | Uncultured Blastococcus | 0.84±0.06 | 1.24±0.05 |
| **OTU1583** | Firmicutes | Clostridia | *Terrisporobacter* | 1.62±0.19 | 0.17±0.01 |
| **OTU1173** | Proteobacteria | Alphaproteobacteria | *Skermanella* | 0.81±0.01 | 0.98±0.02 |
| **OTU214** | Chloroflexi | KD4-96 | KD4-96 norank | 0.64±0.06 | 1.05±0.05 |
| OTU1449 | Acidobacteria | Acidobacteria | Acidobacteria norank | 0.80±0.03 | 0.84±0.05 |
| OTU1006 | Actinobacteria | Actinobacteria | *Microlunatus* | 0.74±0.03 | 0.83±0.08 |
| OTU107 | Proteobacteria | Deltaproteobacteria | *Archangium gephyra* | 0.68±0.04 | 0.81±0.04 |
| **OTU1425** | Proteobacteria | Betaproteobacteria | Nitrosomonadaceae norank | 0.56±0.04 | 0.92±0.09 |
| **OTU1916** | Acidobacteria | Acidobacteria | Unclassified Acidobacteria | 0.83±0.03 | 0.60±0.02 |
| OTU439 | Proteobacteria | Betaproteobacteria | *Ramlibacter* | 0.67±0.05 | 0.69±0.04 |
| OTU633 | Actinobacteria | Actinobacteria | *Marmoricola* | 0.68±0.01 | 0.60±0.07 |
| OTU167 | Acidobacteria | Acidobacteria | Acidobacteria norank | 0.58±0.04 | 0.70±0.01 |
| **OTU1898** | Actinobacteria | Actinobacteria | *Aeromicrobium* | 0.74±0.06 | 0.51±0.04 |
| OTU1491 | Acidobacteria | Acidobacteria | Acidobacteria norank | 0.63±0.06 | 0.56±0.02 |
| **OTU1310** | Firmicutes | Clostridia | Unclassified Peptostreptococcaceae | 1.00±0.10 | 0.18±0.02 |
| **OTU850** | Actinobacteria | Actinobacteria | Actinobacteria norank | 0.45±0.06 | 0.72±0.01 |
| **OTU1668** | Bacteroidetes | Cytophagia | Cytophagaceae norank | 0.79±0.05 | 0.38±0.02 |
| OTU980 | Proteobacteria | Betaproteobacteria | Nitrosomonadaceae norank | 0.60±0.08 | 0.54±0.02 |
| OTU157 | Chloroflexi | Gitt-GS-136 | Gitt-GS-136 norank | 0.52±0.05 | 0.62±0.03 |
| **OTU629** | Proteobacteria | Betaproteobacteria | *Piscinibacter* | 0.43±0.01 | 0.69±0.04 |
| **OTU1395** | Chloroflexi | Thermomicrobia | JG30-KF-CM45 norank | 0.50±0.02 | 0.60±0.01 |
| **OTU1472** | Proteobacteria | Gammaproteobacteria | *Lysobacter* | 0.62±0.04 | 0.45±0.04 |
| **OTU1456** | Gemmatimonadetes | Gemmatimonadetes | Gemmatimonadaceae norank | 0.50±0.01 | 0.56±0.01 |
| **OTU989** | Firmicutes | Clostridia | *Clostridium butyricum* | 0.97±0.06 | 0.09±0.01 |
| **OTU675** | Actinobacteria | Actinobacteria | *Nocardioides* | 0.44±0.03 | 0.59±0.04 |
| OTU1915 | Actinobacteria | Actinobacteria | *Gaiella* | 0.48±0.03 | 0.54±0.04 |
| **OTU215** | Actinobacteria | Actinobacteria | *Gaiella* | 0.33±0.01 | 0.60±0.02 |
| OTU834 | Actinobacteria | Actinobacteria | Gaiellales norank | 0.38±0.05 | 0.53±0.05 |
| **OTU1069** | Proteobacteria | Alphaproteobacteria | *Bradyrhizobium* | 0.55±0.02 | 0.31±0.01 |
| **OTU810** | Proteobacteria | Gammaproteobacteria | *Lysobacter* | 0.55±0.01 | 0.30±0.01 |
| **OTU433** | Proteobacteria | Betaproteobacteria | *Azohydromonas* | 0.19±0.01 | 0.63±0.01 |
| **OTU942** | Proteobacteria | Gammaproteobacteria | Unclassified Xanthomonadaceae | 0.51±0.01 | 0.30±0.02 |
