# Supplementary material for: Changes in rhizosphere microbial communities in potted cucumber seedlings treated with syringic acid
Source: PLoS One. 2018 Jun 28;13(6):e0200007. doi: 10.1371/journal.pone.0200007 (PMC6023137; doi:10.1371/journal.pone.0200007)
Supplement: S2 Table — OTUs were delineated at the 97% sequence similarity. Only OTUs with average relative abundances >0.1% in at least one treatment were presented. Values were expressed as mean±standard error. OTU ID in bold indicates its relative abundance was significant different between treatments according to Welch’s t test (P<0.05). (DOC) [file pone.0200007.s004.doc]

**Table S2.** **The most abundant fungal OTUs in the syringic acid (SA)- and water (W)-treated soil samples.** OTUs were delineated at the 97% sequence similarity. Only OTUs with average relative abundances >0.1% in at least one treatment were presented. Values were expressed as mean±standard error. OTU ID in bold indicates its relative abundance was significant different between treatments according to Welch’s *t* test (P<0.05).

| OTU ID | Phylum | Class | Genus/Species | Relative abundances (%) | |
| --- | --- | --- | --- | --- | --- |
|  |  |  |  | Water | SA |
| **OTU68** | Ascomycota | Sordariomycetes | Unclassified Chaetomiaceae | 29.58±1.04 | 8.17±0.44 |
| **OTU48** | Ascomycota | Leotiomycetes | Unclassified Thelebolaceae | 6.29±0.63 | 17.94±1.11 |
| **OTU71** | Zygomycota | Zygomycetes | Unclassified Mortierellaceae | 15.85±1.60 | 3.61±0.13 |
| **OTU322** | Ascomycota | Sordariomycetes | Unclassified Lasiosphaeriaceae | 9.63±0.27 | 2.70±0.20 |
| **OTU193** | Ascomycota | Sordariomycetes | *Gibberella* | 1.94±0.23 | 6.93±0.72 |
| **OTU352** | Ascomycota | Sordariomycetes | Unclassified Lasiosphaeriaceae | 8.00±0.35 | 0.17±0.01 |
| **OTU324** | Ascomycota | Sordariomycetes | *Pseudallescheria* | 5.98±0.62 | 1.15±0.07 |
| **OTU97** | Zygomycota | Zygomycetes | *Mortierella elongata* | 1.52±0.13 | 5.32±0.87 |
| OTU228 | Ascomycota | Pezizomycetes | *Pseudaleuria* | 2.19±0.37 | 3.96±2.34 |
| OTU270 | Ascomycota | Pezizomycetes | Unclassified Pyronemataceae | 2.17±0.22 | 3.72±1.36 |
| OTU212 | Ascomycota | Sordariomycetes | *Fusarium* | 0.43±0.06 | 5.12±2.28 |
| **OTU150** | Ascomycota | Sordariomycetes | *Humicola* | 0.61±0.20 | 4.64±0.67 |
| OTU33 | Ascomycota | Pezizomycetes | *Cephaliophora tropica* | 1.35±0.06 | 3.47±1.34 |
| OTU287 | Ascomycota | Pezizomycetes | Unclassified Pyronemataceae | 2.69±0.89 | 1.99±1.05 |
| OTU184 | Unclassified Fungi | Unclassified Fungi | Unclassified Fungi | 2.32±0.64 | 2.30±0.31 |
| **OTU333** | Ascomycota | Sordariomycetes | Unclassified Chaetomiaceae | 0.31±0.04 | 2.25±0.04 |
| **OTU79** | Basidiomycota | Tremellomycetes | *Cryptococcus aerius* | 0.34±0.06 | 2.15±0.24 |
| OTU34 | Ascomycota | Sordariomycetes | *Humicola* | 0.23±0.01 | 2.02±0.45 |
| OTU118 | Zygomycota | Zygomycetes | *Mortierella ambigua* | 0.02±0.00 | 1.55±0.61a |
| **OTU271** | Zygomycota | Zygomycetes | *Mortierella polygonia* | 0.99±0.12 | 0.54±0.11 |
| **OTU181** | Zygomycota | Zygomycetes | *Mortierella* | 0.00±0.00 | 1.34±0.12 |
| **OTU136** | Ascomycota | Sordariomycetes | *Fusarium solani* | 0.25±0.03 | 1.07±0.19 |
| OTU45 | Ascomycota | Sordariomycetes | *Colletotrichum coccodes* | 0.03±0.01 | 0.89±0.26 |
| **OTU323** | Zygomycota | Zygomycetes | *Mortierella* | 0.89±0.15 | 0.02±0.02 |
| OTU74 | Ascomycota | Leotiomycetes | Unclassified Thelebolaceae | 0.30±0.07 | 0.61±0.31 |
| OTU99 | Ascomycota | Dothideomycetes | Unclassified Sporormiaceae | 0.61±0.18 | 0.28±0.01 |
| OTU94 | Ascomycota | Sordariomycetes | *Scedosporium prolificans* | 0.04±0.00 | 0.80±0.47 |
| **OTU206** | Zygomycota | Zygomycetes | *Mortierella alpina* | 0.10±0.01 | 0.74±0.03 |
| OTU307 | Ascomycota | Dothideomycetes | *Preussia flanaganii* | 0.44±0.07 | 0.37±0.02 |
| **OTU164** | Basidiomycota | Tremellomycetes | *Guehomyces pullulans* | 0.05±0.02 | 0.62±0.13 |
| **OTU64** | Ascomycota | Sordariomycetes | *Humicola nigrescens* | 0.11±0.00 | 0.51±0.05 |
| **OTU109** | Ascomycota | Sordariomycetes | *Wardomyces inflatus* | 0.07±0.01 | 0.54±0.07 |
| **OTU173** | Ascomycota | Sordariomycetes | Unclassified Microascaceae | 0.12±0.02 | 0.45±0.04 |
| OTU326 | Ascomycota | Sordariomycetes | *Gibberella* | 0.15±0.04 | 0.42±0.08 |
| **OTU13** | Ascomycota | Sordariomycetes | *Chaetomium aterrimum* | 0.07±0.01 | 0.46±0.04 |
| OTU171 | Zygomycota | Zygomycetes | *Modicella reniformis* | 0.00±0.00 | 0.51±0.15 |
| OTU86 | Zygomycota | Zygomycetes | *Mortierella alpina* | 0.05±0.01 | 0.46±0.11 |
| OTU81 | Zygomycota | Zygomycetes | *Mortierella hyalina* | 0.09±0.01 | 0.39±0.15 |
| OTU319 | Basidiomycota | Agaricomycetes | *Coprinellus canistri* | 0.46±0.27 | 0.00±0.00 |
| **OTU24** | Ascomycota | Eurotiomycetes | Unclassified Onygenaceae | 0.00±0.00 | 0.44±0.10 |
| **OTU318** | Ascomycota | Sordariomycetes | *Myrothecium verrucaria* | 0.12±0.03 | 0.30±0.03 |
| **OTU215** | Ascomycota | Sordariomycetes | *Fusarium brachygibbosum* | 0.06±0.01 | 0.36±0.01 |
| **OTU18** | Ascomycota | Eurotiomycetes | Unclassified Onygenaceae | 0.11±0.03 | 0.30±0.04 |
| OTU73 | Ascomycota | Dothideomycetes | *Curvularia* | 0.00±0.00 | 0.39±0.24 |
| OTU273 | Ascomycota | Sordariomycetes | *Kernia pachypleura* | 0.03±0.01 | 0.36±0.08 |
| **OTU170** | Ascomycota | Sordariomycetes | *Kernia pachypleura* | 0.03±0.01 | 0.35±0.04 |
| OTU77 | Ascomycota | Sordariomycetes | *Podospora* | 0.16±0.02 | 0.20±0.04 |
| OTU294 | Ascomycota | Sordariomycetes | Unclassified Lasiosphaeriaceae | 0.20±0.03 | 0.09±0.00 |
| OTU202 | Unclassified Fungi | Unclassified Fungi | Unclassified Fungi | 0.00±0.00 | 0.28±0.12 |
| **OTU143** | Ascomycota | Dothideomycetes | Pleosporales incertae sedis | 0.03±0.01 | 0.26±0.04 |
| OTU296 | Ascomycota | Dothideomycetes | *Preussia terricola* | 0.21±0.04 | 0.07±0.01 |
| **OTU144** | Ascomycota | Sordariomycetes | *Monographella cucumerina* | 0.07±0.02 | 0.19±0.02 |
| **OTU125** | Ascomycota | Sordariomycetes | *Chaetomium atrobrunneum* | 0.05±0.01 | 0.21±0.02 |
| **OTU351** | Ascomycota | Eurotiomycetes | *Gymnoascus reesii* | 0.07±0.00 | 0.18±0.02 |
| OTU349 | Ascomycota | Sordariomycetes | *Chaetomidium gallecicum* | 0.23±0.06 | 0.02±0.01 |
| OTU303 | Ascomycota | Sordariomycetes | *Cercophora* | 0.21±0.10 | 0.02±0.01 |
| **OTU358** | Ascomycota | Sordariomycetes | *Chaetomium nigricolor* | 0.04±0.01 | 0.19±0.01 |
| OTU284 | Ascomycota | Dothideomycetes | Unclassified Sporormiaceae | 0.09±0.02 | 0.13±0.02 |
| OTU224 | Ascomycota | Sordariomycetes | Unclassified Sordariomycetes | 0.00±0.00 | 0.21±0.13 |
| **OTU189** | Ascomycota | Sordariomycetes | Unclassified Hypocreales | 0.04±0.01 | 0.16±0.01 |
| OTU113 | Ascomycota | Sordariomycetes | Unclassified Lasiosphaeriaceae | 0.09±0.01 | 0.10±0.01 |
| OTU20 | Basidiomycota | Agaricomycetes | *Minimedusa polyspora* | 0.00±0.00 | 0.18±0.12 |
| OTU137 | Ascomycota | Sordariomycetes | *Stachybotrys chartarum* | 0.04±0.01 | 0.14±0.03 |
| **OTU195** | Ascomycota | Sordariomycetes | Unclassified Lasiosphaeriaceae | 0.02±0.01 | 0.15±0.01 |
| **OTU248** | Ascomycota | Dothideomycetes | Unclassified Sporormiaceae | 0.04±0.02 | 0.11±0.02 |
| OTU160 | Unclassified Fungi | Unclassified Fungi | Unclassified Fungi | 0.01±0.00 | 0.14±0.05 |
| OTU174 | Ascomycota | Sordariomycetes | *Acremonium alcalophilum* | 0.01±0.00 | 0.14±0.08 |
| OTU238 | Ascomycota | Sordariomycetes | *Chaetomium* | 0.02±0.01 | 0.12±0.04 |
| OTU200 | Ascomycota | Sordariomycetes | *Kernia pachypleura* | 0.01±0.00 | 0.10±0.02 |
| OTU274 | Zygomycota | Zygomycetes | *Mortierella reticulata* | 0.10±0.03 | 0.01±0.00 |
